# Impairments in contractility and cytoskeletal organisation cause nuclear defects in nemaline myopathy

**DOI:** 10.1007/s00401-019-02034-8

**Published:** 2019-06-19

**Authors:** Jacob A. Ross, Yotam Levy, Michela Ripolone, Justin S. Kolb, Mark Turmaine, Mark Holt, Johan Lindqvist, Kristl G. Claeys, Joachim Weis, Mauro Monforte, Giorgio Tasca, Maurizio Moggio, Nicolas Figeac, Peter S. Zammit, Heinz Jungbluth, Chiara Fiorillo, John Vissing, Nanna Witting, Henk Granzier, Edmar Zanoteli, Edna C. Hardeman, Carina Wallgren-Pettersson, Julien Ochala

**Affiliations:** 1 Centre for Human and Applied Physiological Sciences, School of Basic and Medical Biosciences, Faculty of Life Sciences and Medicine, Guy’s Campus, King’s College London, London, SE1 1UL UK; 2 Neuromuscular and Rare Diseases Unit, Department of Neuroscience, Fondazione IRCCS Ca’ Granda Ospedale Maggiore Policlinico, Milan, 20122 Italy; 3 Department of Cellular and Molecular Medicine, University of Arizona, Tucson, AZ 85721 USA; 4 Division of Biosciences, University College London, Gower Street, London, WC1E 6BT UK; 5 Randall Centre for Cell and Molecular Biophysics, School of Basic and Medical Biosciences, Faculty of Life Sciences and Medicine, Guy’s Campus, King’s College London, London, SE1 1UL UK; 6 Department of Neurology, University Hospitals Leuven, Herestraat 49, 3000 Louvain, Belgium; 7 Laboratory for Muscle Diseases and Neuropathies, Department of Neurosciences, KU Leuven, Louvain, Belgium; 8 Institute of Neuropathology, RWTH Aachen University Hospital, Aachen, Germany; 9 Unità Operativa Complessa di Neurologia, Fondazione Policlinico Universitario A. Gemelli IRCCS, Largo A. Gemelli 8, 00168 Rome, Italy; 10 Department of Paediatric Neurology, Neuromuscular Service, Evelina’s Children Hospital, Guy’s and St Thomas’ Hospital National Health Service Foundation Trust, London, SE1 9RT UK; 11 Department of Basic and Clinical Neuroscience, Institute of Psychiatry, Psychology and Neuroscience, King’s College, London, SE1 1UL UK; 12 IRCCS Gaslini and Department of Neuroscience, Rehabilitation, Ophthalmology, Genetics, Maternal and Child Health, Genoa University, Genoa, Italy; 13 Copenhagen Neuromuscular Center, Rigshospitalet, University of Copenhagen, 2100 Copenhagen, Denmark; 14 Department of Neurology, Faculdade de Medicina (FMUSP), Universidade de São Paulo, São Paulo, Brazil; 15 Neuromuscular and Regenerative Medicine Unit, School of Medical Sciences, University of New South Wales, Sydney, NSW 2052 Australia; 16 The Folkhälsan Institute of Genetics and Department of Medical and Clinical Genetics, Biomedicum Helsinki, University of Helsinki, Helsinki, Finland

**Keywords:** Skeletal muscle, Nemaline myopathy, Microtubules, Actin, Lamin, Nuclear envelope

## Abstract

Nemaline myopathy (NM) is a skeletal muscle disorder caused by mutations in genes that are generally involved in muscle contraction, in particular those related to the structure and/or regulation of the thin filament. Many pathogenic aspects of this disease remain largely unclear. Here, we report novel pathological defects in skeletal muscle fibres of mouse models and patients with NM: irregular spacing and morphology of nuclei; disrupted nuclear envelope; altered chromatin arrangement; and disorganisation of the cortical cytoskeleton. Impairments in contractility are the primary cause of these nuclear defects. We also establish the role of microtubule organisation in determining nuclear morphology, a phenomenon which is likely to contribute to nuclear alterations in this disease. Our results overlap with findings in diseases caused directly by mutations in nuclear envelope or cytoskeletal proteins. Given the important role of nuclear shape and envelope in regulating gene expression, and the cytoskeleton in maintaining muscle fibre integrity, our findings are likely to explain some of the hallmarks of NM, including contractile filament disarray, altered mechanical properties and broad transcriptional alterations.

**Electronic supplementary material:**

The online version of this article (10.1007/s00401-019-02034-8) contains supplementary material, which is available to authorized users.

## Introduction

Nemaline myopathy (NM) is a genetically heterogeneous disease of skeletal muscle caused by mutations in genes that are generally involved in muscle contraction, in particular those related to the structure and/or regulation of the thin filament. Mutations in *ACTA1* (skeletal muscle actin) or *NEB* (nebulin) together make up the majority of cases, whilst other causative genes (to date, *TPM3*, *TPM2*, *TNNT1*, *CFL2*, *KBTBD13*, *KLHL40*, *KLHL41*, *LMOD3*, *MYPN, RYR3* or *MYO18B*) together with unidentified genes, are implicated in the remainder [20, 38]. These mutations result in weakness at the contractile level, while other cellular pathological hallmarks include dense accumulations of proteins known as nemaline rods, arrested muscle fibre growth, impaired fibre type differentiation, and disarray of contractile filaments [20]. However, the underlying mechanisms behind many of these features remain uncertain, even though the mutations affecting thin filament structure and function are likely to be involved [7, 17, 28, 29, 40, 42, 60, 63]. In the present study, we aimed to acquire a clearer understanding of muscle fibre dysfunction in NM by specifically studying nuclei and the related cortical cytoskeleton.

Skeletal muscle fibres are large syncytial cells containing many, often hundreds, of nuclei (termed myonuclei). Sufficient numbers and regular spacing of myonuclei throughout the muscle fibre are a prerequisite for its function, allowing the efficient delivery of gene products to all parts of the cell, with minimal transport distances. Therefore it is thought that each nucleus is responsible for maintaining a certain volume of the muscle fibre, termed the myonuclear domain [5, 32]. The nuclei of skeletal muscle fibres are linked with various cytoskeletal components including non-sarcomeric/cytoplasmic actins, microtubules and intermediate filaments such as desmin. All three of these cytoskeletal networks have been implicated in the spacing and positioning of nuclei in models of skeletal muscle development: microtubules in the initial translocation/spacing of nuclei along the fibre [12, 14, 15, 35], and actin and desmin in their movement to the fibre periphery [45].

At the organelle level, nuclear function and transcription are regulated by a host of external factors; the nuclear envelope acts as a signalling hub that is capable of transducing a range of chemical and mechanical signals to regulate gene expression [18, 34, 58]. The cytoskeleton is known to regulate nuclear shape and morphology via interactions with the nuclear envelope [10, 23], a process which can itself impact on gene transcription, with different morphologies being linked to cell type, function, differentiation, and disease states [10, 23, 62]. Given that the force-generating properties of NM muscle fibres are severely limited, we hypothesised that cytoskeletal components as well as nuclear function, positioning and integrity might be affected in this disease, and possibly contribute to pathology.

Using single muscle fibres from mouse models and NM patients with mutations in *ACTA1* or *NEB*, we found that myonuclei display a range of defects, including irregular spacing and morphology, abnormal nuclear envelope and altered chromatin distribution. We also observed severe disruption within the microtubule, desmin and cytoplasmic (β- and γ-) actin networks, as well as alterations in their anchorage at the nuclear surface. We next sought to investigate the underlying pathological mechanisms, and found that impaired contractile force production is responsible for the nuclear spacing and morphological defects. We further demonstrated the role of a properly organised microtubule network in regulating nuclear shape. Our findings suggest that these alterations are likely to contribute to some of the features observed in NM, which include: broad transcriptional alterations and hindered muscle fibre growth [30, 32] (perhaps due to the nuclear disruption which is likely to affect gene expression programmes); myofibril disarray (since desmin and the nuclear envelope are known to contribute to their organisation [1, 6]); and altered mechanical properties of muscle fibres (known to be related in part to the cortical cytoskeleton) [21]. In addition, our results suggest that nuclear and cytoskeletal defects might be a secondary feature and/or source of pathology in other (muscle) diseases, even in those where these structures are not primarily affected.

## Materials and methods

### Human subjects

All tissue was consented, stored, and used in accordance with the Human Tissue Act, UK, under local ethical approval (REC 13/NE/0373). Details of patients providing samples for light microscopy are given in Table 1, and of patients for electron microscopy in Tables 2 and 3. No electron microscopy samples were available from the patients used for light microscopy studies, hence, the different cohorts of patients for the two techniques.

**Table 1:** Nemaline myopathy patient muscle biopsy samples used for light microscopy

Gene	Age	M/F	Mutation (DNA)	Mutation (protein)	Source
*ACTA1*	20	M	c.16G>A	E6K	Copenhagen, Denmark
*ACTA1*	30	F	c.841T>C	Y281H	Genoa, Italy
*NEB*	36	F	c.2836-2A>G and c.5763+5G>A	Mutation in splice site	Copenhagen, Denmark
*NEB*	56	M	c.17234C>T and c.2271_22713del	R5745X and K7571del	Copenhagen, Denmark
*NEB*	71	F	c.508-7T>A and c.19097G>T	Mutation in splice site; and S6366I	Helsinki, Finland

Age at biopsy is given (years)

**Table 2:** Nemaline myopathy patient muscle biopsy samples used for electron microscopy

Gene	Age	M/F	Mutation (DNA)	Mutation (protein)	Source
*NEB*	2 (a)	M	c.17737-2A>T and c.21315delA	Mutation in splice site and R7105 frameshift	Milan, Italy
*NEB*	2 (b)	M	c.4082+5G>T (splice site) and g.112388C>T	Mutation in splice site and Q>X	Milan, Italy
*NEB*	17	M	c.22249A>C and c.8392-8395 duplication	T7417P and R2799L frameshift	Helsinki, Finland
*NEB*	23	M	c.11164C>T and c.19097G>T	R3722* (nonsense) and S6366I	Helsinki, Finland
*NEB*	30	F	c.508-7T>A and c.19097G>T	Mutation in splice site and S6366I	Helsinki, Finland
*ACTA1*	11 weeks	F	c.796T>C	F226L	London, UK
*ACTA1*	3	M	c.235A>G	T79A	Milan, Italy
*ACTA1*	10	M	c.841T>C	Y281H	Milan, Italy

Age at biopsy is given (years, unless otherwise specified)

**Table 3:** Patients with sporadic late-onset nemaline myopathy (SLONM), used for electron microscopy

Patient	Age	Age of onset	M/F	Monoclonal gammopathy	Source
SLONM 1	65	64	M	IgG lambda	Rome, Italy
SLONM 2	69	68	F	Not available	Rome, Italy
SLONM 3	72	67	F	No	Leuven, Belgium
SLONM 4	79	54	F	Not available	Leuven, Belgium
SLONM 5	34	32	F	No	Leuven, Belgium

Ages at biopsy/examination and age of onset are shown in years. SLONM 1 and 2 correspond to patients 4 and 6 in Monforte et al. [36]; SLONM 3, 4 and 5 correspond to patients 2, 5, and 6 in Schnitzler et al. [53]. All patients were HIV-negative

### Mouse models

*Acta1*^H40Y^ mouse tibialis anterior muscle samples, with and without transfer of the *Myl4* gene remained frozen from a previous study [30]. To summarise, data were collected from four wild type and four mutant male mice. At 4 weeks of age, the compartment containing the tibialis anterior muscle was injected with rAAV6 virus containing the *Myl4* transgene, and contralateral legs served as controls, being injected with virus lacking the functional gene. Mice were sacrificed 4 weeks later at 8 weeks of age. Colony maintenance and experiments were approved by the Uppsala Local Ethical Committee on Animal Research.

*Neb* cKO mice [28] were maintained at the University of Arizona in accordance with the US National Institutes of Health guidelines “Using Animals in Intramural Research”. Mutants and WT/heterozygous littermates were sacrificed at 3 months of age by cardiac perfusion with 4% paraformaldehyde (PFA)/PBS, to properly preserve microtubule structure. Extensor digitorum longus muscles were dissected for whole-mount immunolabelling of cytoplasmic (β- and γ-) actins, desmin or microtubules (see below).

### Antibodies

Primary antibodies were as follows (species, isotype, manufacturer, catalogue number, and dilution are given): lamin A (mouse monoclonal IgG3, Abcam, ab8980, 1:200); nesprin-1 (rabbit monoclonal IgG, Abcam, ab192234, 1:400); pericentrin (rabbit polyclonal IgG, Abcam, ab4448, 1:200); β-tubulin (clone TUB2.1, mouse monoclonal IgG1, Santa Cruz, sc-58886, 1:500); desmin (clone D33, mouse monoclonal IgG1, Dako, M076001-2, 1:400); β-actin (rabbit polyclonal IgG, Abcam, ab8227, 1:300); γ-actin (clone 2A3, mouse monoclonal IgG2b, Bio-Rad, MCA5776GA, 1:300); acetyl lys9/lys14 histone H3 (rabbit polyclonal IgG, cell signaling, #9677, 1:200).

### Enzymatic isolation and culture of intact single muscle fibres

Intact single muscle fibres were prepared as described previously, using enzymatic dissociation with collagenase I (Sigma Aldrich) and gentle trituration (Sigma Aldrich) [47]. After isolation, fibres were plated into 6-well plates (~ 30 fibres per well) in DMSO/high glucose/GlutaMAX supplement/pyruvate (Thermo Fisher Scientific, Cat# 31966021), containing 10% horse serum and 1% penicillin/streptomycin solution. Freshly isolated fibres were treated overnight with nocodazole (20 μM), taxol (10 μM) or epothilone D (10 μM). Final DMSO (vehicle) concentration was 0.5% in all cases. To assess myonuclear spacing, fibres were cultured for 72 h in the presence of the aforementioned drugs.

### Immunohistochemistry (single muscle fibres)

Myofibres were fixed in 4% PFA/PBS for 15 min, and washed 3× in PBS. Fibres were permeabilised in 0.1% triton-X/PBS for 10 min, washed 3× and blocked in 10% normal goat serum/PBS for 1 h. Fibres were then treated with primary antibodies in blocking solution overnight (β-tubulin) or for 3 h (lamin A, nesprin-1, pericentrin) at 4 °C. Fibres were washed 3× in PBS for a total of 30 min, and then treated with Alexa 594 or 488-conjugated secondary antibodies and DAPI (all at 1:1000 in PBS) for 3 h. Fibres were washed 3× in PBS for a total of 30 min and mounted in Fluoromount mounting medium (Southern Biotech) with coverslip (thickness #1.5).

### Immunohistochemistry (whole-mount muscles)

*Neb* cKO mice and WT/heterozygous littermates (all female) were sacrificed at 3 months of age by cardiac perfusion with 4% PFA/PBS, to properly preserve microtubule structure. Extensor digitorum longus muscles were dissected for whole-mount immunolabelling of cytoplasmic (β- and γ-) actins, desmin or microtubules. Muscles were then permeabilised in 0.5% triton-X/PBS (20 min) and 0.1% Triton-X/PBS (20 min), with each solution being replaced at least once during the incubation. Samples were then blocked in mouse-on-mouse block/PBS for 3 h, and then blocked in 8% bovine serum albumin overnight. Primary antibodies (tubulin, cytoplasmic actins) in blocking buffer were applied for 5 h, followed by 2 h washing in 0.1% triton-X/PBS.

### Fluorescence imaging

Fibres were imaged on a Zeiss Axiovert 200 spinning disc confocal microscope equipped with BD CARV II and a motorised Z drive at 20× magnification (for imaging of nuclear morphology and nuclear envelope). For nuclear number and distribution, Z-stacks with 1 μm Z increments were taken through the entire depths of fibres, as described previously [48, 49]. For imaging of cytoskeleton and nuclear volume, a Nikon A1 laser scanning confocal microscope with a 100× oil immersion objective (1.4 NA) was used, with Z-stacks taken with 0.3 μm Z increments (Nikon Imaging Centre, King’s College London).

### Image analysis

Analysis of nuclear number and spacing: Coordinates of myonuclei were identified in 3D within Z-stacks of muscle fibres. A custom-made Matlab programme was used to a measure fibre CSA, nuclear number, nearest neighbour distances and order score (‘g’) of nuclei within fibres, as described previously [4, 48].

Analysis of nuclear shape parameters: For 2- and 3-dimensional measurements (area, aspect ratio, circularity, and volume), nuclei in the DAPI channel were thresholded by pixel intensity until fully highlighted. Inbuilt ImageJ functions were used to measure 2D parameters and Voxel Counter plugin for volume. For accurate shape analysis, nuclei positioned on the sides or the backs of fibres (relative to the microscope objective) were excluded, as were those in clusters where nuclei were touching.

Microtubule quantifications: density (% area) was calculated on binary converted images in ImageJ. Microtubule directionality was calculated using the TeDT tool [31].

### Statistics

Graphs were prepared and analysed in Graphpad Prism. Linear regression lines and statistical comparisons were performed using inbuilt algorithms (ANCOVA test was used to compare elevations/intercepts and slopes of different regression lines). For statistical comparisons of nuclear organisation and nuclear shape in human subjects (column graphs), control data points were pooled, since no significant age-related differences were observed amongst healthy control subjects. Owing to the different origins of disease (mutation and gene affected), patient data points were not pooled. One-way ANOVA with Tukey post-correction was used to compare each patient against the pooled controls; in addition, a random effect algorithm was incorporated into the model, to account for any potential inter-individual differences that might exist amongst the control cohort. For studies with animals, no significant differences were observed between animals of the same genotype, and as such, individual data points for both control and mutant/treated animals were pooled. For column comparisons in the animal studies, a two-tailed *t* test was used to compare two groups, and a one-way ANOVA with Tukey post-correction was used to compare more than two groups. For drug treatments with nocodazole, taxol, and epothilone D in ex vivo culture experiments, a cumulative probability test (Kolmogorov–Smirnov test) was used to compare groups against control/vehicle only. Asterisks denote the following statistical significance levels: *P < 0.05), **P < 0.01, ***P < 0.001.

## Results

### Muscle fibres from NM patients have misshapen and mispositioned nuclei with altered chromatin organisation

**Fig. 1:**
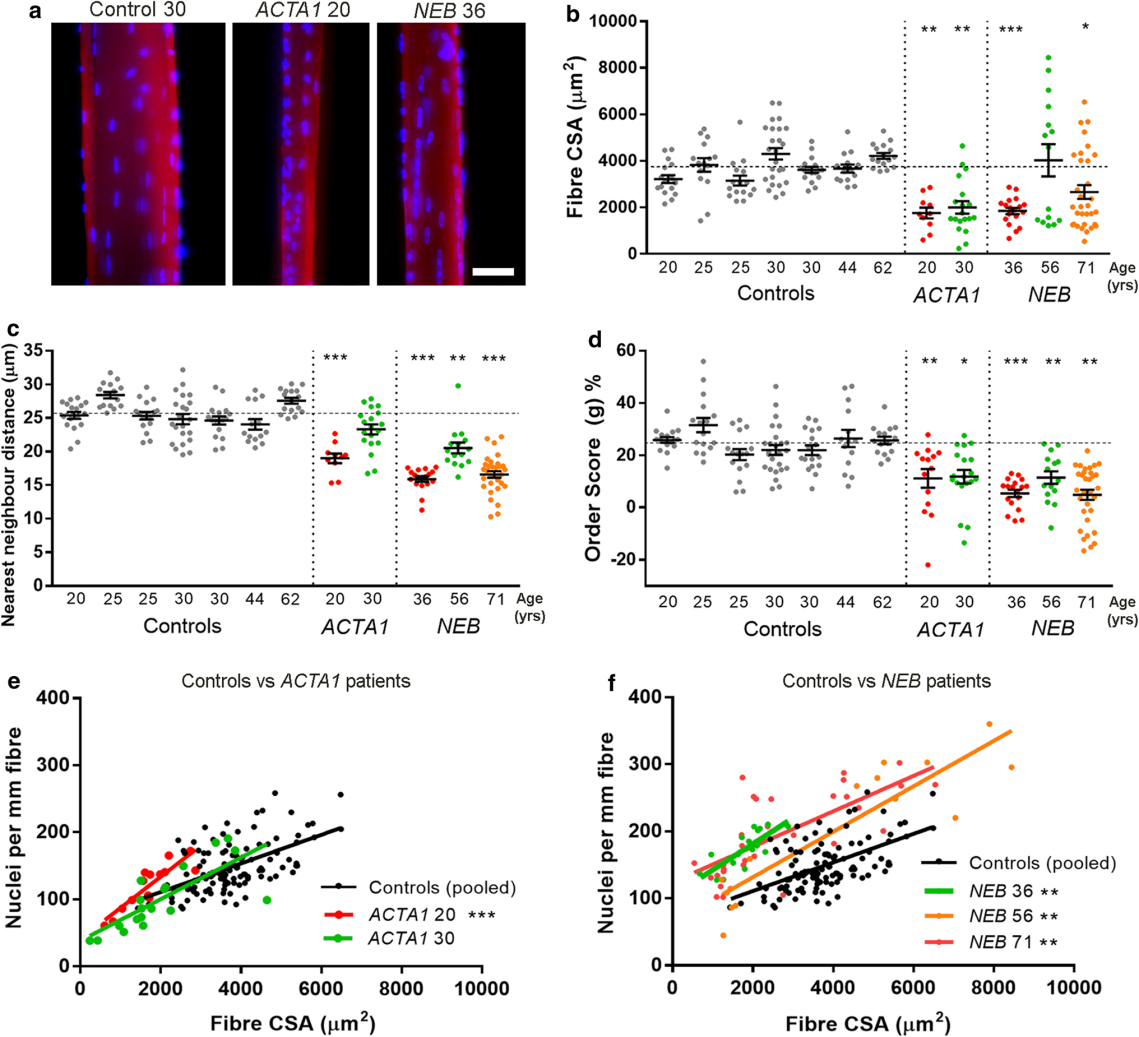
Myonuclei are more abundant and unevenly spaced in patients with nemaline myopathy. Healthy control subjects and patients are denoted with their mutation and age. **a** Representative single skeletal muscle fibres from a control subject and patients with *ACTA1* or *NEB* mutations, labelled for actin (rhodamine phalloidin; red) and nuclei (DAPI; blue). **b** Fibre cross-sectional area (CSA) for controls and patients. **c** Nearest neighbour distance between myonuclei within fibres; a smaller distance indicates greater density of nuclei. **d** Order score (g), an algorithm to assess the regularity of nuclear spacing [4]; a lower score indicates more irregular spacing and more nuclear clustering. **e** Comparison between controls and *ACTA1* patients: relationship between number of nuclei per mm of fibre length, and their CSA. **f** As for **e**, but comparing controls and *NEB* patients (graphs **e** and **f** separated for visual clarity). For regression lines, data points from control subjects were pooled since there were no significant inter-individual variations (full data available in Fig. S1a, b). Individual data points represent an individual skeletal muscle fibre, with mean ± SEM, or linear regression lines. For column graphs, significance was determined using one-way ANOVA comparing each patient with a group consisting of all control data points pooled together (since no significant inter-individual variation was found amongst controls). ANCOVA was used to compare elevations/intercepts and slopes of regression lines. Scale bar: 50 μm. *P < 0.05, **P < 0.01, ***P < 0.001

To assess myonuclear distribution in patients with NM, single skeletal muscle fibres were teased from biopsy samples, mounted, and labelled with rhodamine-labelled phalloidin to visualise actin (thus marking the dimensions of the fibre) and DAPI to label nuclei, and imaged in 3D. Ages of controls were as follows: 20, 25, 25, 30, 30, 44, and 62. Ages of *ACTA1* patients were 20 and 30, and ages of *NEB* patients were 36, 56, and 71 (patient details in Table 1). Muscle fibres were generally smaller in cross-sectional area (CSA) in patients compared with age-matched control subjects, a common characteristic of NM (as well as some other muscle disorders), although some fibres in *NEB* patients (ages 56 and 71) displayed large CSA values, indicating some extent of fibre size disproportion (Fig. 1a, b). In one *ACTA1* patient (aged 20) and all three *NEB* patients, there was a higher abundance of myonuclei within muscle fibres compared to control subjects, as observed in nearest neighbour distances (Fig. 1c; lower values indicating more densely packed nuclei), and in numbers of nuclei per length of fibre (Fig. 1e, f; across a range of fibre sizes/CSAs; linear regression lines were similar for all control subjects as shown in Suppl Fig. S1a, b (online resource); hence for visual clarity, control data were pooled in these graphs).

Order score, a measure of the regularity of nuclear spacing, was also calculated [4]. Mean order score was significantly lower in all patients compared with control subjects, indicating that nuclei were more unevenly distributed than in controls (Fig. 1d). No correlation between fibre CSA and order score was noted in most controls and patients, indicating that nuclear distribution was not noticeably affected by fibre size (Suppl Fig. S1c, d). Central nuclei are a hallmark of certain neuromuscular disorders including muscular dystrophies; however, this was rare in NM patient fibres, with the vast majority of nuclei being located at the fibre periphery, as expected. Overall, these results suggest that myonuclear positioning is altered in NM patients, and that there is often a higher density of myonuclei within fibres.

**Fig. 2:**
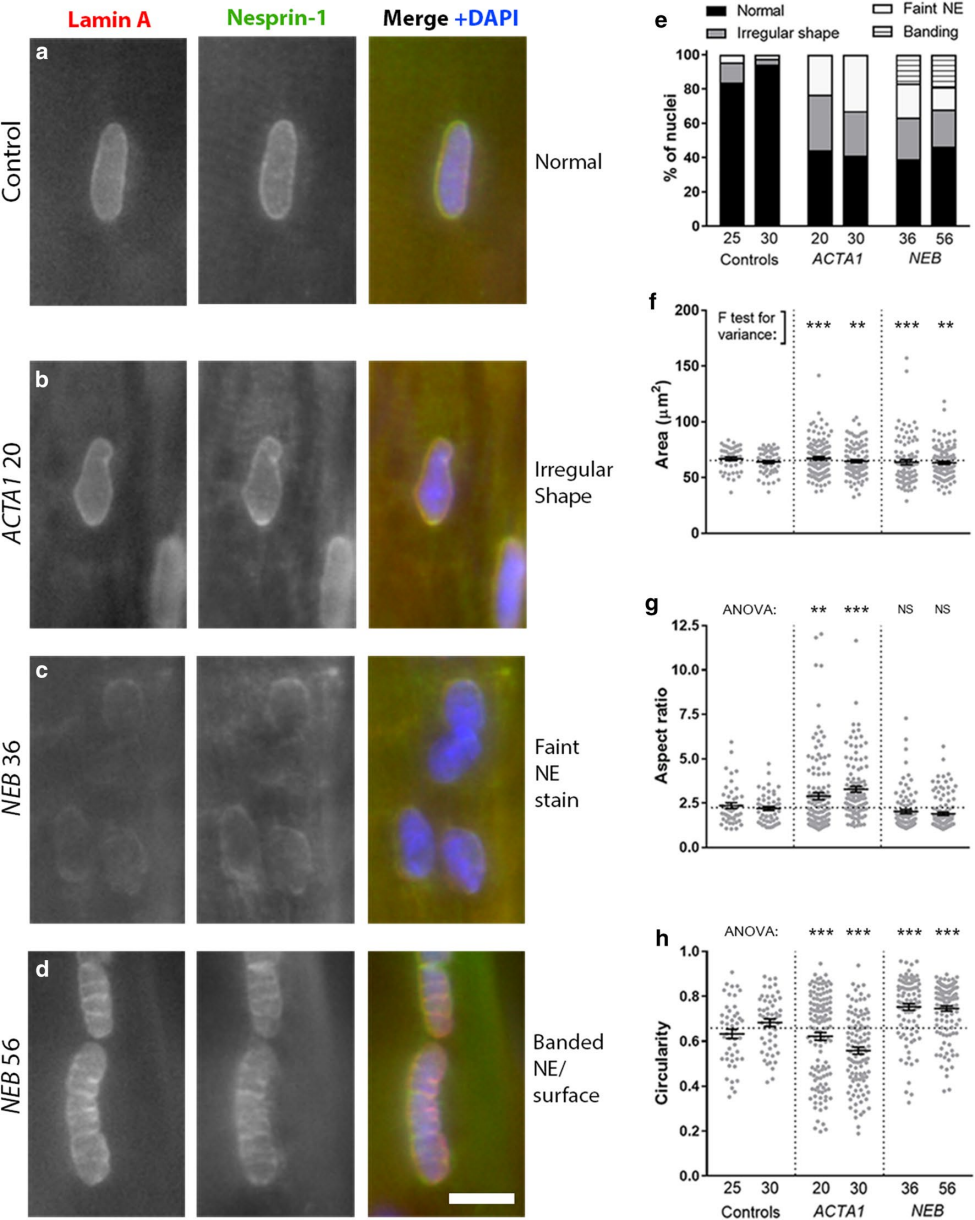
Nuclear morphology and nuclear envelope are altered in skeletal muscle fibres of nemaline myopathy patients. Healthy control subjects and patients are denoted with their mutation and age. **a**–**d** Representative images of myonuclei from healthy controls and NM patients with *ACTA1* or *NEB* mutations, immunolabelled for lamin A (left panels), nesprin-1 (middle panels) and merged with DAPI (Lamin A, green; nesprin-1, red; DAPI blue). Myonuclei displaying **a** normal shape and nuclear envelope; **b** irregular shape; **c** faint immunolabelling for lamin A and nesprin-1; **d** banded pattern across surface. Graph showing proportions of each nuclear phenotype for patients and two controls (**e**). Graphs showing shape quantifications for nuclei: area (**f**), aspect ratio (**g**) and circularity (**h**). A larger aspect ratio denotes a more elongated shape; a higher circularity denotes a more circular shape. Graphs (**f**–**h**) show one data point per nucleus analysed, taken from > 10 muscle fibres per subject, with mean ± SEM. For graph (**f**), an *F* test for variance was used, to report differences in spread/variability of each patient versus controls; for graphs **g**, **h**, one-way ANOVA was used to report differences in mean between each patient and a group consisting of both control subjects pooled together. Note differences in overall data distribution as well as mean values. Scale bar: 10 μm. *NS* not significant *P < 0.05), **P < 0.01), ***P < 0.001

Given that the distribution of nuclei was perturbed in NM patient muscle fibres, we next wanted to determine if there were any alterations within the nuclei themselves. In control subjects (aged 25 and 30), nuclei generally possessed an elliptical shape, and nuclear envelope proteins nesprin-1 and lamin A localised in a regular rim around the nuclear edges, as expected (Fig. 2a, control subject). However, in the two *ACTA1* patients (ages 20 and 30) and two *NEB* patients (ages 36 and 56) studied, > 50% of the myonuclei were altered in each case, displaying various features including shape irregularities (Fig. 2b), faint immunolabelling of nuclear envelope proteins (Fig. 2c), and a banded appearance across the nuclear surface (Fig. 2d). Varying proportions of these features were observed in the four patients analysed (Fig. 2e). Nuclear shape/elongation was further analysed using area (in the 2D *X*–*Y* plane), aspect ratio and circularity measures (Fig. 2f–h). Although the nuclear area was on average similar in controls and patients, the variability was significantly greater in patients (Fig. 2f). Nuclei in *ACTA1* patients shifted towards a more elongated shape (larger aspect ratio, lower circularity), and nuclei in *NEB* patients towards a more circular shape (lower aspect ratio, higher circularity; Fig. 2g, h). Similar nuclear defects are observed in laminopathies, which are primary genetic disorders of the nuclear envelope, and include muscular dystrophies and multisystem disorders caused by mutations in lamin A/C, nesprins and other genes [19]. Our results indicate that severe defects in nuclear envelope and morphology can also occur in diseases caused by muscle contractile dysfunction.

**Fig. 3:**
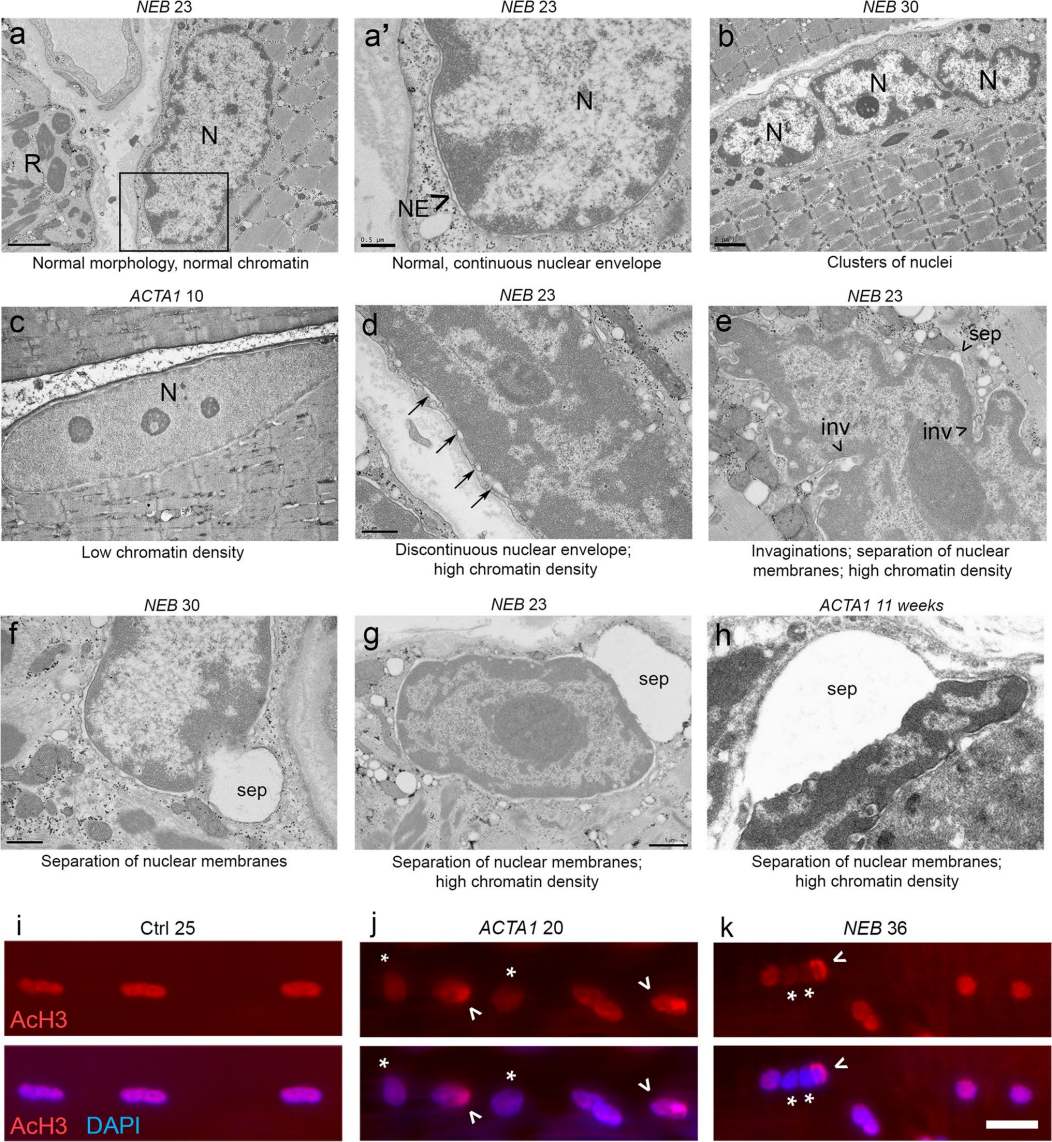
Nuclear envelope and chromatin alterations in myonuclei of nemaline myopathy patients. Electron micrographs of patient muscle tissue, denoted with their mutation and age. **a**, **a′** An example of an apparently relatively normal nucleus (N), with normal morphology, chromatin distribution, and a nuclear envelope consisting of a continuous double membrane marked “NE” (**a′** is a magnified image of the boxed region in **a**). Nemaline rods (R) are seen in an adjacent fibre (**a**). The following nuclear abnormalities were also observed in patients: clusters of nuclei (**b**); reduced chromatin density (**c**); increased chromatin density (**d**, **e**, **g**, **h**); discontinuous patches of nuclear envelope (**d**, arrows); invaginations (**e**, denoted “inv”); and separation of the two nuclear membranes (**e–h**, denoted “sep”). Width of separation between inner and outer nuclear membranes in **e**–**h** is 0.16, 0.89, 1.89, and 2.16 μm, respectively, (compared to apparently normal areas with separations measuring 25–40 nm (typical of normal nuclear envelope). **i**–**k** Skeletal muscle fibres were immunolabelled with antibody against acetylhistone H3 (Lys9/Lys14), a marker of transcriptionally active regions of DNA (red). DNA is stained with DAPI (blue). Myonuclei from control subjects showed an even distribution of staining throughout the nuclei; however, in patients, some nuclei showed reduced fluorescence intensity, possibly indicative of global transcriptional downregulation within certain nuclei (asterisks); and uneven distribution of staining, suggesting irregular packing of active genomic regions (arrowheads). Similar results were observed in one other control subject (age 30), and one other *ACTA1* and *NEB* patient (ages 30 and 56, respectively). 50+ nuclei were observed per subject across ~ 9 fibres for light microscopy. Quantifications of acetylhistone H3 immunolabelling are found in Suppl Fig. S2 (online resource), and semi-quantitative analysis of electron microscopy findings in Table 4. Scale bar (**i**–**k**): 20 μm

Next, ultrastructural imaging was carried out, revealing nuclear morphological and envelope defects in more detail (Fig. 3; see Table 2 for patient details). In NM patients, a proportion of myonuclei resembled those seen in healthy muscle tissue, being of normal shape, having a regular distribution of heterochromatin largely at the nuclear periphery, and with a nuclear envelope consisting of a continuous double membrane (Fig. 3a, a′). However, various defects were also noted: clusters of nuclei (Fig. 3b); low chromatin density (i.e., high levels of euchromatin; Fig. 3c); high chromatin density (i.e., high levels of heterochromatin; Fig. 3d, e, g, h); discontinuities in the nuclear envelope (arrows, Fig. 3d); invaginations (marked ‘inv’, Fig. 3e); regions of separation between inner and outer nuclear membranes (marked ‘sep’, Fig. 3e–h). Interestingly, in the latter cases, chromatin was never observed to fill the open regions, again suggesting that the abnormality was the result of separations between the two membranes, rather than breakage or disruption of both. Table 4 gives a semi-quantitative analysis of the patients studied, listing the various abnormal features and the relative frequency of occurrence. Fluorescence microscopy of acetylhistone H3 (Lys9/Lys14), a well-characterised marker of transcriptionally active regions of DNA [16, 24, 44], provided further evidence of chromatin redistribution within nuclei of patients relative to control subjects (Fig. 3i–k; image quantifications in Suppl Fig. S2, online resource). These results demonstrate alterations in the nuclear envelope and in chromatin organisation, which would be expected to affect nuclear function and transcriptional regulation.

**Table 4:** Ultrastructural observations in myonuclei of nemaline myopathy patients

Patient (mutation/age)	Clusters of myonuclei	Chromatin density	Invaginations	Separation of inner and outer nuclear membranes	
*NEB* 2 (a)	N	↑	+	N	
*NEB* 2 (b)	N	↓↓	+	N	
*NEB* 17	+	↓↓	++	N	
*NEB* 23	++	↑	+	++	~ 50% nuclei with separations ranging ~ 0.15–0.2 μm; 3 nuclei with separations ranging 0.84–1.89 μm (34 nuclei studied in total)
*NEB* 30	+	↑	+	+	3 nuclei with separations ranging 0.89–1.44 μm (25 nuclei studied in total)
*ACTA1* 11 weeks	Only one nucleus image recorded			+	Separation of 2.16 μm
*ACTA1* 3	N	↓↓	N	N	
*ACTA1* 10	N	↓↓	N	N	

Categorisation is based on criteria described in Fig. 3. For chromatin density, ↑ denotes an increase in density (more heterochromatin) and ↓ a decrease in density (less heterochromatin). + indicates the presence of a given feature/observation. N denotes none observed. In all cases, two symbols indicate a particularly high incidence whereby the majority of observed myonuclei displayed the characteristic in question. 6–34 myonuclei observed in all patients, across multiple fibres and fields of view, unless otherwise specified. Expected distance between inner and outer nuclear membranes is 20–40 nm, which was confirmed by our own measurements in regions of nuclei that appeared normal

### Reduced cellular force production is responsible for myonuclear alterations in NM

**Fig. 4:**
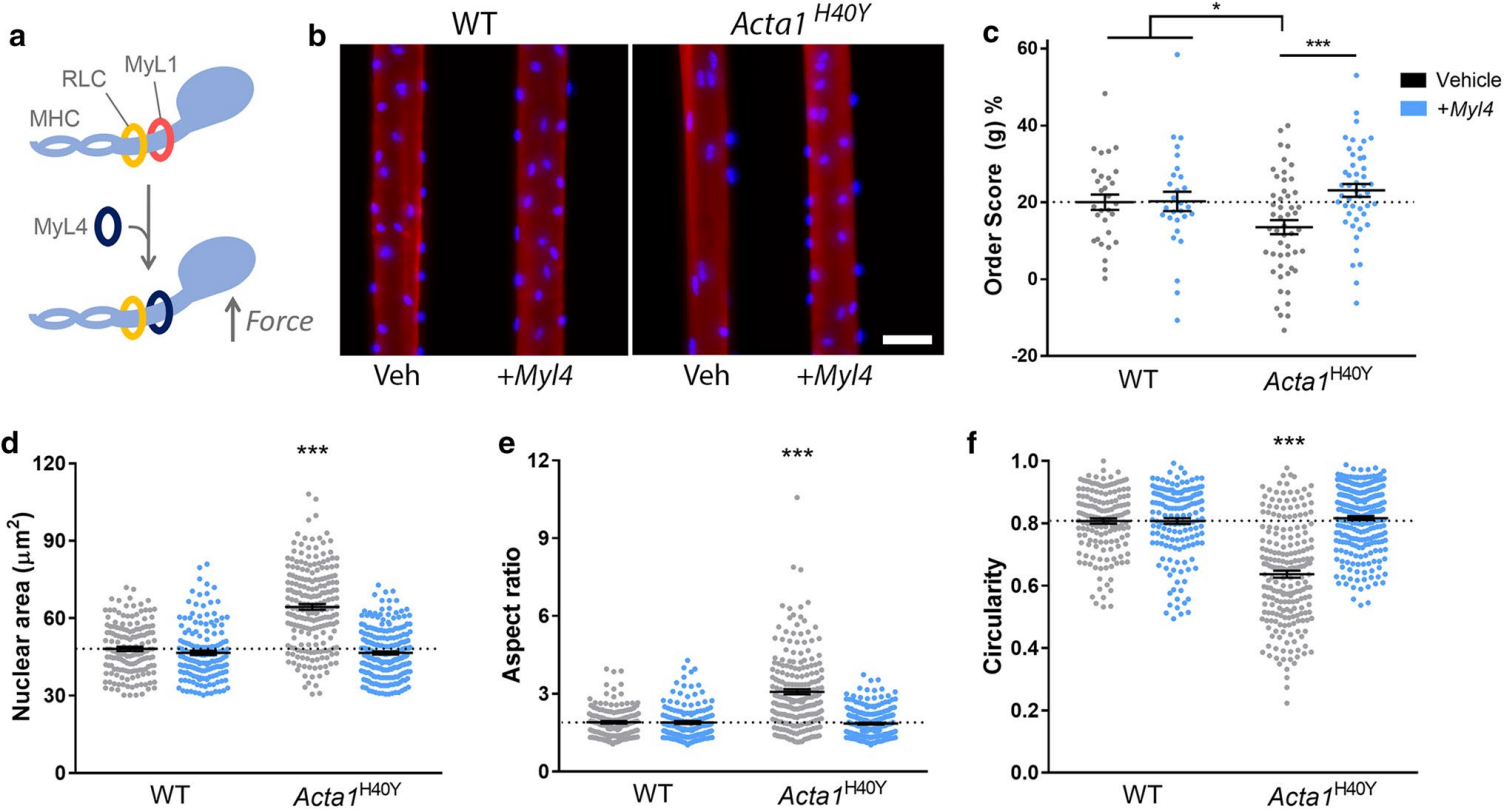
Partial rescue of force production results in full restoration of nuclear spacing and morphology in the *Acta1*^H40Y^ model of nemaline myopathy. **a** Scheme for mouse model published previously, where a gene therapy approach was used to deliver a transgene (*Myl4*) into tibialis anterior muscles [30]. *Myl4* encodes a myosin light chain isoform (MyL4) normally only expressed in developing muscles, but when incorporated into the adult myosin complex, results in a myosin with increased force production (MHC, myosin heavy chain; RLC, regulatory light chain; MyL1, the endogenous myosin light chain). **b** Representative single skeletal muscle fibres from wild type (WT) and *Acta1*^H40Y^ mice, treated with empty vector (Veh), or *Myl4* transgene. Fibres were stained for actin (rhodamine phalloidin; red) and nuclei (DAPI; blue). **c** Order score (g), an algorithm to assess the regularity of nuclear spacing [4]; A lower score indicates more irregular spacing and more nuclear clustering. Nuclear shape measurements: nuclear area as observed in standard *x*, *y* planes (**d**), aspect ratio (**e**) and circularity (**f**). Note in **c**–**f** that the alterations in nuclear spacing and morphology observed in mutants were restored to wild-type levels when treated with *Myl4* transgene. **c** One data point per muscle fibre; **d**–**f** one data point per nucleus analysed, mean ± SEM, with asterisks denoting significance versus WT/vehicle (one-way ANOVA). Scale bar: 50 μm. *P < 0.05, **P < 0.01, ***P < 0.001

To investigate potential mechanisms behind aberrant nuclear spacing and morphology in NM, we employed a severe model of the disease, the *Acta1*^H40Y^ mouse [29, 37, 59]. In a recently published study, a gene therapy approach was used to enhance myosin force output, via the delivery of a myosin light chain isoform (MyL4) that is normally only expressed in developing skeletal muscles (schematic, Fig. 4a). This resulted in a partial rescue of the *Acta1*^H40Y^ phenotype, including an improvement in muscle force production and an increase in muscle fibre size [30]. Thus, we aimed to determine whether an increase in force output could also rescue the nuclear defects observed in NM. Like patients with *ACTA1* or *NEB* mutations, skeletal muscle fibres of *Acta1*^H40Y^ mice showed an irregular distribution of myonuclei (Fig. 4b), which was quantifiable using the order score parameter (Fig. 4c). However, delivery of the *Myl4* transgene resulted in the full restoration of nuclear spacing defects to wild-type levels (Fig. 4c). In addition, nuclear shape alterations, including increased area and aspect ratio and reduced circularity (Fig. 4d–f), were also restored to wild-type values in muscles of *Acta1*^H40Y^ mice treated with the transgene. These results suggest that in NM, force impairment results in nuclear spacing and morphological alterations, and that enhancing force production restores these parameters.

To gain further insight into whether force impairments affect nuclear structure in human subjects, we studied electron microscopy images of five patients with sporadic late-onset nemaline myopathy (SLONM). This disease contrasts with typical NM cases, since it is an acquired condition that occurs in the absence of mutations in known disease-related genes. However, like NM of known genetic origin, on the histopathological level this disease presents with nemaline rods, sarcomeric disarray, and crucially, reduced capacity of muscle to generate force. Patient details are given Table 3, and were published in two recent studies [36, 53] (ages: 34, 65, 69, 72, 79). A number of nuclear defects were observed in all five patients, and recapitulated those observed in our analysis (Fig. 3) of patients with *ACTA1* or *NEB* mutations. These included nuclear invaginations (Suppl Fig. S3a, b, online resource); reduced accumulation of heterochromatin (Suppl Fig. S3a); increased accumulation of heterochromatin (Suppl Fig. S3b, d, e); and separations between inner and outer nuclear membranes (Suppl Fig. S3c-e). A semi-quantitative analysis of these observations is shown in Table 5. These results indicate that nuclear defects also occur in presumably non-genetic phenocopies of NM, and also provide some correlative evidence that a reduction in force-generating capacity of muscle might underlie these defects (although other aspects of myofibre pathology might also contribute).

**Table 5:** Ultrastructural observations in myonuclei of patients with sporadic late-onset nemaline myopathy (SLONM, an acquired form of the disease)

Patient (age)	Clusters of myonuclei	Chromatin density	Invaginations	Separation of inner and outer nuclear membranes	
SLONM 1 (65)	+	↑↑, some ↓	++	+	2 nuclei with separations of > 2 μm (15 nuclei studied in total)
SLONM 2 (69)	N	↑	+	N	
SLONM 3 (72)	+	↑↑	+	+	2 nuclei with separations 0.38 and 0.85 μm (14 nuclei studied in total)
SLONM 4 (79)	+	↑↑, some ↓	+	N	
SLONM 5 (34)	N	↑↑	++	N	

See Table 3 for patient details, and recently published studies [36, 53]. Categorisation is based on criteria described in Fig. 3 and Suppl Fig. S3 (online resource). For chromatin density, ↑ denotes an increase in density (more heterochromatin) and ↓ a decrease in density (less heterochromatin). + indicates the presence of a given feature/observation. N denotes none observed. In all cases, two symbols indicates a particularly high incidence whereby the majority of observed myonuclei displayed the characteristic in question. 6–15 myonuclei observed in all patients, across multiple fibres and fields of view. Expected distance between inner and outer nuclear membranes is 20–40 nm, which was confirmed by our own measurements in regions of nuclei that appeared normal

### Muscle fibres from NM patients and mouse models have disrupted cytoskeleton networks

**Fig. 5:**
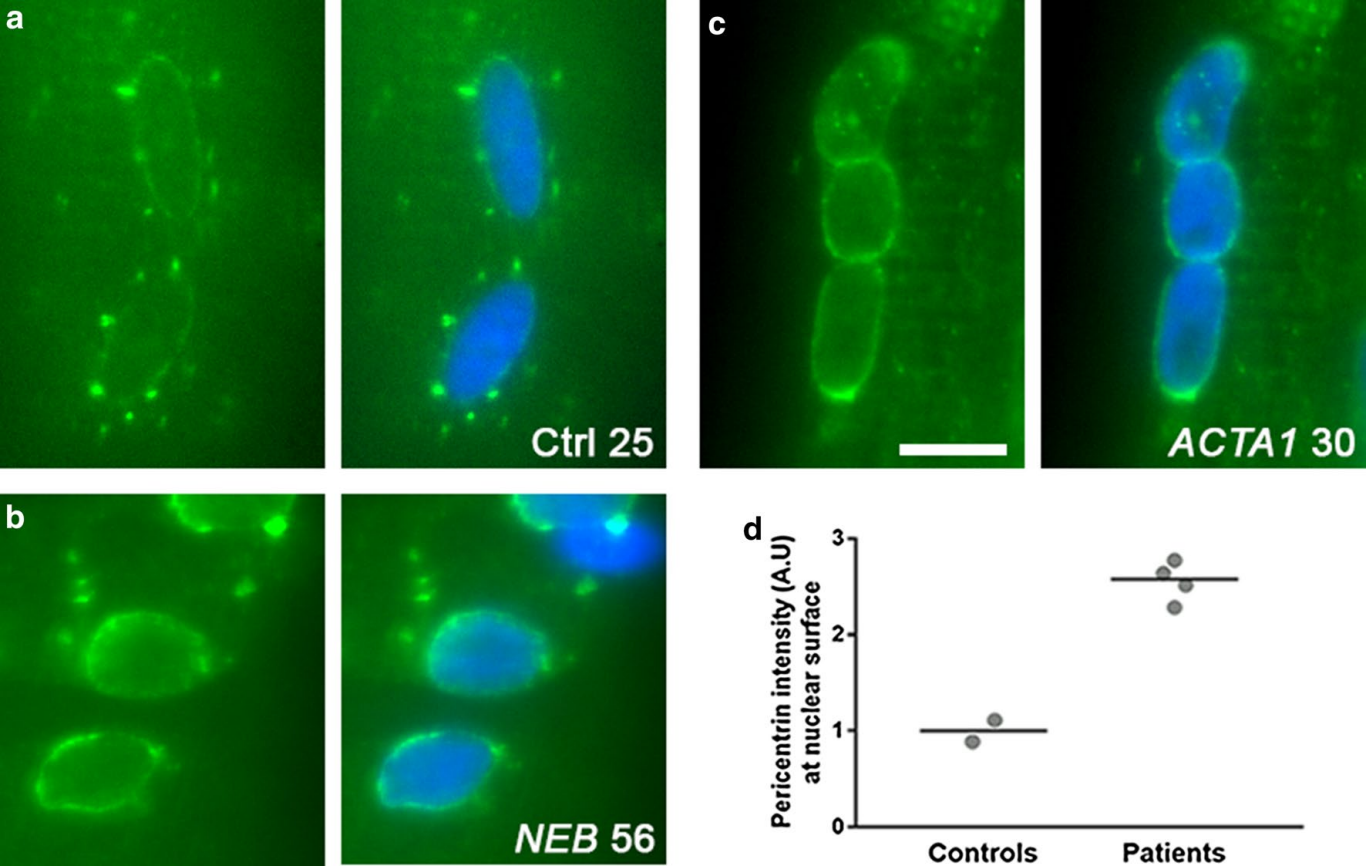
Distribution of the microtubule-organising centre around myonuclei of nemaline myopathy patients. Healthy control subjects and patients are denoted with their mutation and age. **a**–**c** Representative images of nuclei immunolabelled for pericentrin (green) and DAPI (blue). Note specific arrangement of pericentrin in controls (nuclear surface and puncta) versus patients (marked accumulation at the nuclear surface). **d** Quantification of fluorescence intensity of pericentrin at the nuclear surface (within 2 μm of nearest nuclear (DAPI)-labelled pixel). Quantification includes controls aged 25 and 30, two *ACTA1* patients (20 and 30) and two *NEB* patients (36 and 56). Scale bar: 10 μm

In studies using cultured myotubes (an in vitro analogue of muscle fibre formation) and in developing *Drosophila*, the lateral spacing/positioning of myonuclei is known to be dependent on microtubules and motor proteins including kinesins and dyneins [1, 12, 14, 35]. In addition, these motor proteins regulate nuclear shape during their translocation across nascent *Drosophila* fibres [14]. Given that we observe defects in nuclear morphology and spacing in NM patients, we sought to determine whether the microtubule network and/or its associated proteins were perturbed. Microtubules are sensitive to temperature and rapidly depolymerise when tissue is kept cold or frozen [61], and therefore preservation of their structure is not compatible with routine human muscle biopsy preparation. As a surrogate, we aimed to analyse the localisation of pericentrin, a key microtubule organising centre protein (MTOC) that in skeletal muscle is found at the perinuclear regions, consistent with the role of the myonucleus as a nucleator of microtubules. Using the same control subjects and patients from Fig. 2, We found that the localisation of pericentrin was markedly altered in NM patient myonuclei (Fig. 5a–d; note the rim of pericentrin around the nuclear surface, as well as nearby bright puncta in control subjects; note the increased accumulation of pericentrin immunolabelling at the nuclear surface in patients).

**Fig. 6:**
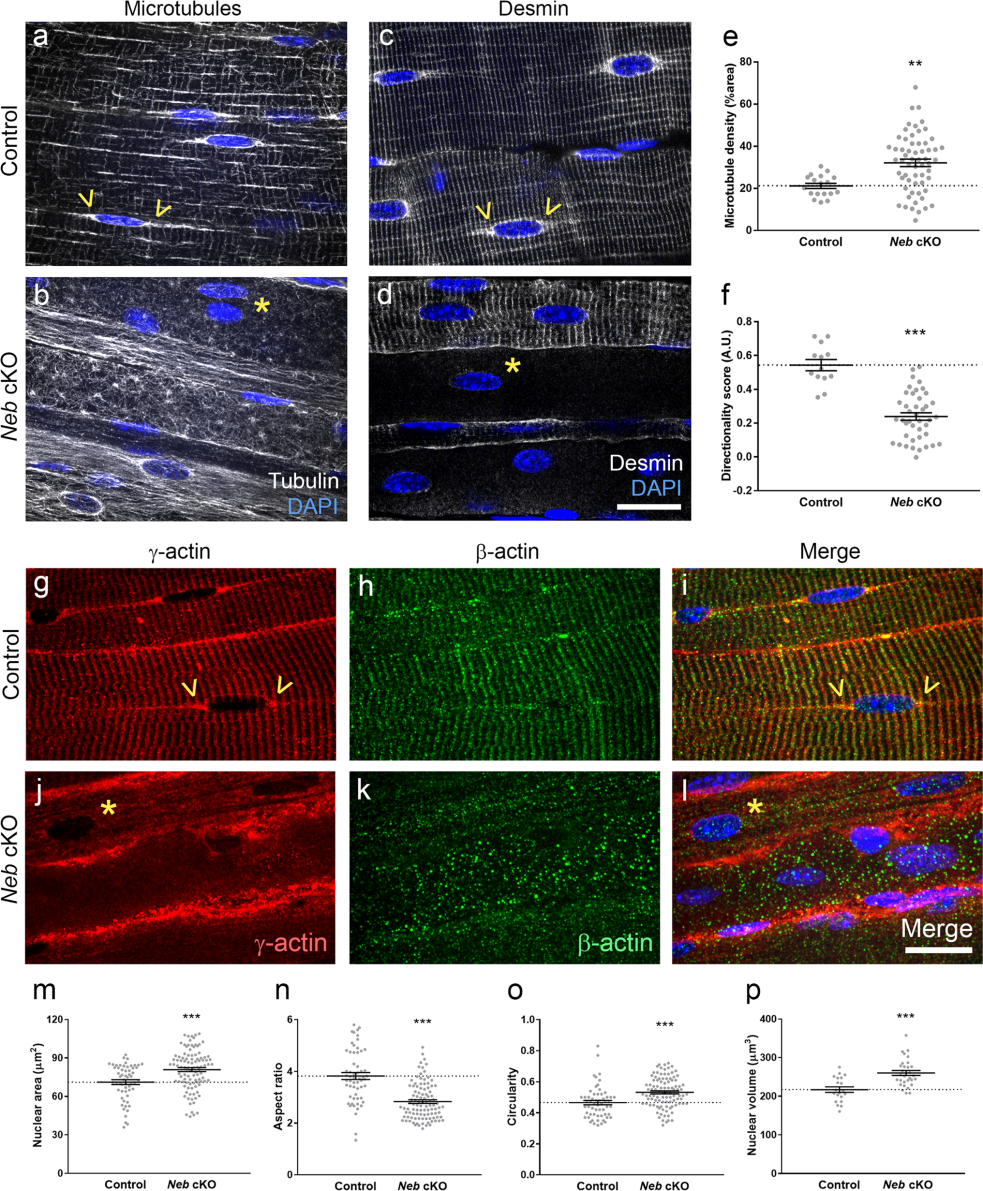
Severe disruption of the cortical cytoskeleton in the *Neb* cKO mouse model of nemaline myopathy. Representative confocal micrographs of skeletal muscle fibres in whole-mount extensor digitorum longus muscles of control (**a**, **c**) and *Neb* cKO mice (**b**, **d**); tissue was immunolabelled with antibodies to β-tubulin (**a**, **b**) or desmin (**c**, **d**) and nuclei were stained with DAPI (blue). In control muscles, the skeletal muscle fibre cortex shows a regular grid-like pattern for microtubules and a striated appearance for desmin; both are largely disorganised or reduced in mutants. Note the clustering of microtubules and desmin at the periphery of nuclei, often altered or lacking in mutants. Quantifications of microtubule density (**e**), and directionality using previously developed algorithms [31] (**f**). Immunolabelling for cytoplasmic (β- and γ-) actins, for control (**g**–**i**) and Neb cKO (**j**–**l**) mice; γ-actin (**g**, **j**), β-actin (**h**, **k**) and merged images (**i**, **l**). Note that microtubules, desmin and γ-actin show specific accumulation at the surface and poles of nuclei (arrowheads), which is frequently reduced or missing in mutants (asterisks). Observations were similar in three mutants and three control littermates. Graphs showing quantifications of nuclear shape parameters in muscle fibres: nuclear area (**m**), aspect ratio (**n**), circularity (**o**) and volume (**p**). Scale bars: 20 μm. Graphs, mean ± SEM, two-tailed *t* test. *P < 0.05), **P < 0.01, ***P < 0.001

To further investigate microtubule organisation, we utilised a mouse model of NM with a conditional knockout in the nebulin gene (*Neb* cKO) [28], and cardiac perfusion fixed with PFA, to allow the proper preservation of microtubule structure. We found that microtubules at the cortex of skeletal muscle fibres were heavily disorganised compared to their control littermates (Fig. 6a, b). Note that the microtubules in control animals appear as a regular grid-like lattice, with denser accumulations around the nuclei (Fig. 6a, arrowheads). In mutants, however, the microtubule network was markedly disorganised, and the extent of accumulation around many of the myonuclei was visibly reduced (Fig. 6b, asterisks). Further analysis indicated that the microtubule network was denser in the mutant animals (Fig. 6e), and that their directionality was disturbed (Fig. 6f; a measurement made using the TeDT algorithm tool developed by Liu and Ralston [31]).

Desmin is a muscle-specific intermediate filament protein that connects myofibrils at the Z-discs, and links to other organelles including nuclei. We found that in most (~ 90%) of *Neb* cKO fibres, the normal Z-disc localisation of desmin was absent, and that its accumulation at the nuclear surface was not discernible (asterisks, Fig. 6c, d). Similar results were observed for cytoplasmic actins (β- and γ-) which form part of the cortical cytoskeleton. In control animals, both β- and γ-actin localised in striations, previously shown to be in alignment with Z-discs of the sarcomere (Fig. 6g–i) [43, 50, 56]. However, in *Neb* cKO animals, both the striations and the nuclear regions of cytoplasmic actins were virtually absent, although γ-actin appeared as accumulations at the fibre periphery (Fig. 6j–l). Together these results indicate that microtubule, desmin and cytoplasmic actin networks are markedly altered in this mouse model of NM.

In addition, myonuclear shape analysis was performed on *Neb* cKO animals (Fig. 6m–p), and as in patients with *NEB* mutations (Fig. 2), nuclei were less elongated/more circular (Fig. 6n, o). In addition, both nuclear area (in 2D) and overall nuclear volume were increased in *Neb* cKO animals compared with controls (Fig. 6m, p).

### Cytoskeletal organisation defines myonuclear properties

**Fig. 7:**
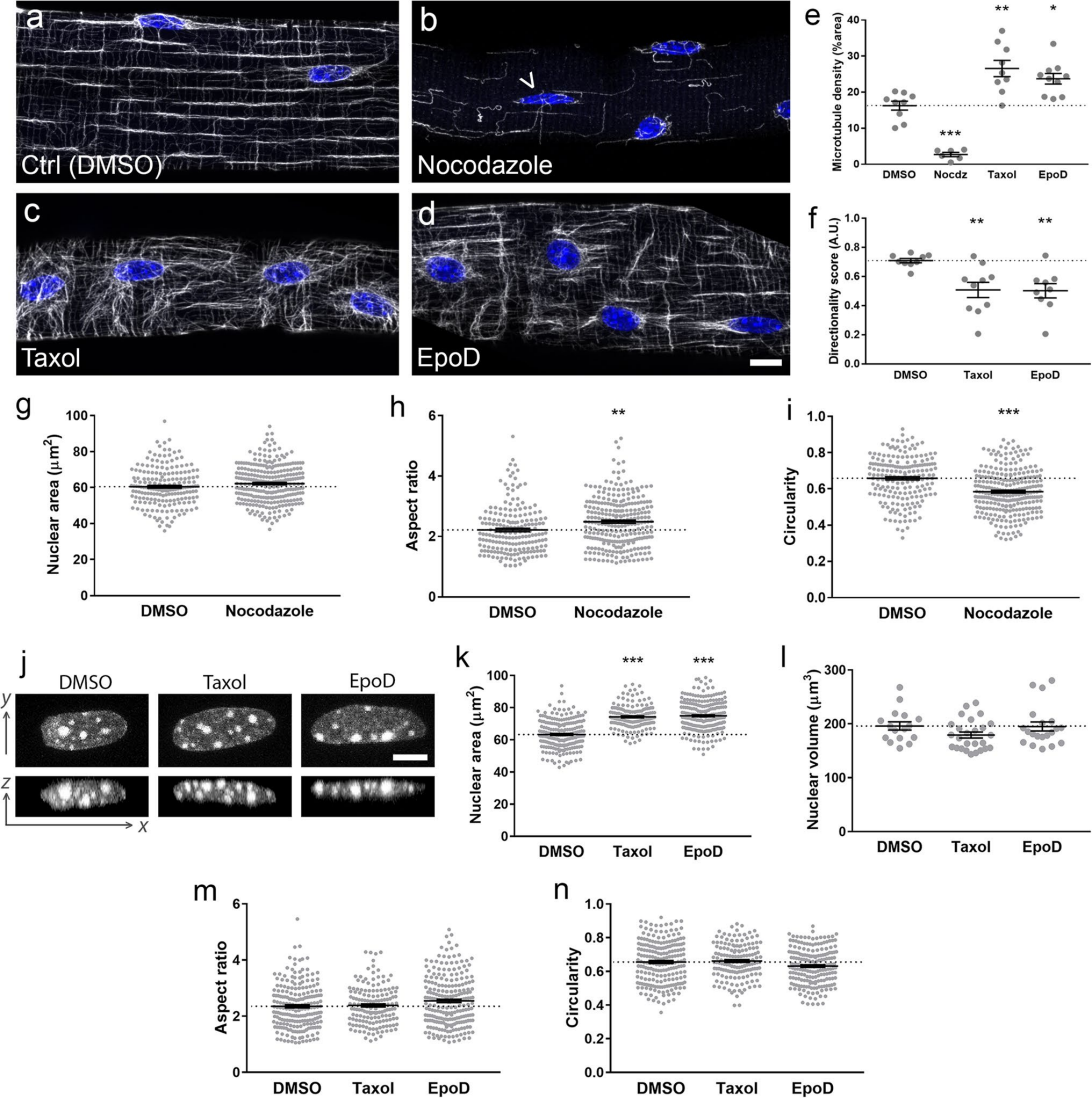
Pharmacological disruption of microtubule network structure results in alterations to nuclear morphology. Enzymatically dissociated single muscle fibres from wild-type mouse extensor digitorum muscles treated overnight with **a** DMSO (control), **b** Nocodazole, **c** taxol and **d** Epothilone D (EpoD). Microtubules are shown in grey (β-tubulin antibody), and nuclei in blue (DAPI). Quantifications of microtubule density (**e**), and directionality using previously developed algorithms [31] (**f**), in drug-treated fibres. Nuclear shape measurements for control versus nocodazole-treated myofibres: nuclear area as observed in 2 dimensions (**g**), aspect ratio (**h**) and circularity (**i**); these results show a modest shift towards more elongated nuclei with nocodazole treatment (arrowhead in B shows example of an elongated nucleus). Representative images of nuclei from myofibres treated with DMSO, taxol and EpoD (**j**): upper row, nuclei as observed in standard *x*, *y* planes; lower row, orthogonal views of nuclei as seen in *x*, *z* planes. Nuclear shape measurements for control versus taxol- and EpoD-treated fibres: nuclear area as observed in standard *x*, *y* planes (**k**), nuclear volume (**l**), aspect ratio (**m**) and circularity (**n**). All graphs show mean ± SEM; one data point per nucleus in panels **g**–**i**, **k**–**n**. Microtubule and nuclear measurements were taken from multiple regions in eight fibres per condition, spread across 2–3 separate experiments for each condition. Asterisks denote significance versus DMSO/control-treated fibres. Scale bars: 10 μm (**a**–**d**) and 5 μm (**j**). Kolmogorov–Smirnov cumulative probability test was used to compare groups. *P < 0.05, **P < 0.01, ***P < 0.001

To investigate whether disorganisation of the cytoskeleton might cause nuclear abnormalities, intact muscle fibres were isolated enzymatically from mouse extensor digitorum longus (EDL) muscles, and cultured with and without drugs that modulate microtubule structure and dynamics. Overnight treatment with nocodazole resulted in an almost complete removal of microtubules (Fig. 7a, b, e), whereas treatment with taxol or Epothilone D (EpoD) resulted in increased microtubule density and reduced directionality score compared with control/vehicle treated fibres (Fig. 7c–f). Nocodazole resulted in small shifts in nuclear morphology towards a more elongated, less circular phenotype (Fig. 7h, i; arrowhead in Fig. 7b indicates an example of an abnormally elongated nucleus), although nuclear area as assessed in 2D was not affected (Fig. 7g). Treatment with taxol or EpoD resulted in an increase in nuclear area (*X*–*Y* planes) compared with control fibres (Fig. 7j, top row of panels; Fig. 7k), although no changes in aspect ratio or circularity were found (Fig. 7m, n). In addition, using 3D Z-stacks, we found similar nuclear volumes between control, taxol- and EpoD-treated fibres (Fig. 7l). This suggests that the increases in nuclear area observed with taxol and EpoD are caused by the nuclei spreading out/becoming flatter, rather than by an overall expansion of their total volume. Consistent with this, the lower panels in Fig. 7j show examples of the Z plane of the Z-stacks (orthogonal views), with nuclei appearing flatter in this dimension. These results imply that the microtubule network exerts tension on the nuclear surface to regulate nuclear flattening.

It should be noted that myonuclear spacing was preserved in all drug-treated fibres relative to control, even after 72 h treatment (Fig. 7a–d and Suppl Fig. S4, online resource). Also, there were no gross changes in nesprin-1 localisation with any of the drug treatments, suggesting that microtubule disruption did not have any major effects on the nuclear envelope (Suppl Fig. S4). These results indicate that at least in non-contracting isolated muscle fibres, microtubule disruption for this length of time has no major impact on nuclear positioning or the nuclear envelope.

## Discussion

In this study, we identify a range of nuclear and cytoskeletal defects in skeletal muscle fibres that occur as part of *ACTA1* or *NEB*-related NM pathology. We show that such defects in nuclear morphology and spacing are caused by the impairment in force production that is a characteristic of this disease. We also highlight the role of the microtubule cytoskeleton in the regulation of nuclear shape.

Myonuclear spacing defects (Fig. 1) have also been observed in other mutant mouse models, including those in genes encoding nuclear envelope proteins (SUN1/SUN2 double KO, Nesprin-1 KO [26, 57, 64]), and also in ageing [5]. Interestingly, this effect appears to be specific, since knockout of other nuclear envelope proteins such as lamin A/C does not significantly alter myonuclear organisation [16, 27]. Currently, there is little insight into whether defects in nuclear spacing contribute to myofibre dysfunction, or whether they are merely a secondary phenomenon. However, given that regular spacing of myonuclei is a highly conserved feature across invertebrates and vertebrates, it is assumed that it is important for proper muscle fibre function, such as inter-nuclear cooperation and the efficient distribution of gene products throughout the cell [32]. Therefore, one might envisage that any deviation from a “normal” nuclear arrangement would result in sub-optimal muscle fibre function.

Studies to ascertain the mechanisms of nuclear spacing in skeletal muscle have largely taken place in myotubes and embryos of *Drosophila* and mouse, and have identified a number of mediators including nesprins, microtubules, MTOC proteins and the motor proteins kinesin and dynein [14, 15, 57]. Myotubes, as an in vitro system, are analogous to the events during embryonic development, whereby myonuclei are located in the centre/core of the fibre, where their spacing takes place along the axis of the fibre. As such, myotubes are not anatomically or developmentally equivalent to mature muscle fibres, in which nuclei are anchored at the fibre periphery. In these examples of NM, it is unclear whether the nuclear organisation defects are a result of aberrant spacing occurring during development, or in events that occur in maturity. However, our results show that nuclear positioning can be remodelled in mature fibres, since delivery of the *Myl4* isoform into adult *Acta1*^H40Y^ mice rescued nuclear spacing defects (Fig. 4). A key feature of muscle tissue in NM (as well as some other myopathies) is a shift towards type I fibres. This alone is unlikely to explain the alterations that we observed in NM patients, since no clear difference in the regularity of nuclear spacing is observed across human fibre types [9].

Various nuclear envelope and shape defects are also a feature of NM patients (Figs. 2, 3). Perhaps associated with this is the frequent observation of chromatin abnormalities in NM patients by EM and light microscopy (Fig. 3, Suppl Fig. S2, online resource), since both nuclear morphology and the nuclear envelope are known to play a role in transcriptional regulation via chemical and mechanical control of chromatin organisation [10, 18, 23, 34, 58, 62]. Given that NM is caused by mutations in genes related to contraction, it would appear that these alterations are a secondary defect. However, many of these alterations bear striking resemblance to those seen in primary diseases of the nuclear envelope, for instance those caused by mutations in genes encoding, e.g., lamins, nesprins, or emerin, which frequently present with muscular dystrophy [2, 13, 19, 33, 52, 55]. Given the severity of disease caused by mutations in genes encoding nuclear envelope proteins, it is likely that the array of nuclear defects that we describe in NM patients contributes to muscle dysfunction. Indeed, broad alterations in the transcriptional profile of skeletal muscle are observed in patients with NM (including in genes related to metabolism and calcium homeostasis), and this may partially result from these characteristic nuclear shape and envelope alterations and/or reorganised chromatin [51]. It should be noted that intranuclear rods are also a feature of some cases of NM, which might also be expected to affect nuclear function; however, we did not observe any instances of these in the patient samples used in this study (either by EM, or by α-actinin staining in light microscopy samples).

Delivery of the *Myl4* transgene to adult *Acta1*^H40Y^ mouse muscles augments contractile force [30] and rescues nuclear spacing and morphology (Fig. 4). This suggests that (i) a lack of force originating at the sarcomere is responsible for these nuclear defects, and that (ii) this can be reversed by an increase in contractile capacity. The mediators of this effect are unclear, but may involve direct force transmission to the nuclei via, e.g., microtubule, actin or desmin cytoskeletons, or interactions through second messengers that may be responsive to mechanical input. Interestingly, nuclear defects at the ultrastructural level were also observed in patients with acquired forms of NM (SLONM; Suppl Fig S3, online resource; Table 5), where a reduction in muscle contractile force also occurs, but in the absence of mutations in known disease-causing genes. This provides some evidence that contractile dysfunction is a cause of nuclear defects in humans, although this data are currently only supportive. In a broader sense, nuclear abnormalities might be common to other neuromuscular diseases, since various other classes of myopathy are also associated with impaired contraction [20]. One example that has been studied in detail is Marinesco–Sjögren syndrome, a multisystem disorder with myopathy, which is caused by mutations that affect endoplasmic reticulum trafficking and chaperone function [25, 46]. Ultrastructural abnormalities in myonuclei of this disorder include highly condensed chromatin and areas of nuclear envelope separation, akin to our observations in nemaline myopathy. It should be noted that disease etiology is highly variable across neuromuscular disorders, and that various aspects of muscle pathology might influence nuclear function and integrity, besides impaired contractile function.

Another of our key findings was the cytoskeletal defects in the *Neb* cKO model NM, involving microtubules, desmin and non-sarcomeric actins (Figs. 5, 6). Microtubules also show increased density and disorganisation in dystrophic mice with dystrophin or sarcoglycan deficiency, or mice with MAP6 ablation [3, 54]. In skeletal muscle, microtubules are known to have roles in modulating fibre stiffness and contraction, and in signalling via reactive oxygen species (ROS) [21]. Indeed, the increased microtubule density in dystrophic mice results in elevated ROS, over-activation of stretch-sensitive Ca^2+^ channels, and worsened pathology [22]. In the mouse model of NM utilised in the present study, defects in non-sarcomeric (β- and γ-) actins and desmin were striking in that the vast majority of fibres showed markedly reduced/virtually absent localisation of all of these components. Ablation of either β- or γ-actin specifically in skeletal muscle causes mild progressive myopathies [39], and loss of desmin causes disruptions of muscle architecture [6]. Intriguingly, myofibrils are frequently misaligned and disordered in NM [60], and this might be due to (i) the loss of desmin, and/or (ii) the defects at the nuclear envelope, since both are involved in the proper arrangement of myofibrils [1, 6]. One key role of desmin and actins is the lateral transmission of force to the fibre periphery, where actin and microtubules bind to the dystrophin-associated glycoprotein complex at the plasma membrane [3, 43, 56]. Therefore, the mislocalisation of these components is likely to have implications for the mechanical properties of the fibre.

Cytoskeletal components also have important roles in anchorage at the nucleus, and the normal localisation of microtubules, γ-actin and desmin at the nuclear surface were also largely reduced in *Neb* cKO mice. The cytoskeleton is likely to transmit strain from the mechanical forces of contraction to the nuclei, which may be important for fibre integrity and/or regulation of gene expression [8]. Consistent with this, disruption of microtubules with several agents resulted in alterations to nuclear shape (Fig. 7). Although microtubules are known to regulate nuclear spacing during development, no changes in nuclear distribution were observed when fibres were treated with nocodazole, taxol or EpoD (Fig. 7; Suppl Fig. S4, online resource). Possible explanations for this include: (i) other cytoskeletal systems are instead responsible for nuclear spacing in mature fibres, or are able to compensate when microtubules are disrupted; (ii) nuclei are more mobile in actively contracting fibres, which was not the case in these experiments; or (iii) longer treatments are required to induce significant remodelling of nuclei (which would not be preferable due to the relatively short-term viability of muscle fibres in ex vivo conditions).

The range of disease severity varies greatly in NM, and death in childhood is frequent at the most severe end of the spectrum. In this study, the patients were almost entirely of adult age, representing the milder end of the spectrum (with the exception of some early-onset cases included for electron microscopy studies). This was an experimental design choice, due to the availability of healthy human control tissue at adult, but not childhood ages. Therefore, it is difficult to draw extensive conclusions regarding nuclei in typical congenital cases of NM, although several early-onset cases displayed invaginations, altered chromatin compaction and in one case, separation of inner and outer nuclear membranes (Fig. 3, Table 4). To date, our understanding of human muscle development at the cellular level during infancy and childhood is incomplete, and is likely to be highly dynamic throughout this period of sustained growth [11]. As such, interpretation of cellular organisation in congenital NM patients would be difficult, without appropriate information on healthy development.

In summary, our results demonstrate that skeletal muscle from NM patients and mouse models display defects in the non-sarcomeric cytoskeleton and in nuclear positioning and integrity. They indicate that abnormal nuclear spacing and morphology are the result of the impaired contractile force production that is a key feature of this disease. In addition, we highlight the role of a properly organised cytoskeleton in the regulation of nuclear morphology. Although nuclear defects are observed in other diseases, including those caused by mutations in nuclear envelope proteins, these findings are somewhat unexpected, given that NM pathology originates at the sarcomere [7, 41]. They might explain some of the features observed in NM, such as broad transcriptional changes and hindered muscle fibre growth (possibly related to alterations in nuclear envelope and chromatin organisation, which is likely to affect programmes of gene expression [18, 34, 62]), myofibrillar disarray (due to the roles of desmin and the nuclear envelope in sarcomere organisation [1, 6]) and altered fibre mechanical properties (due to disrupted cytoskeletal arrangements [21]). This study raises the possibility that nuclear and cytoskeletal defects may be an overlooked feature and/or source of pathology in other (muscle) diseases.

## Electronic supplementary material

Below is the link to the electronic supplementary material.

Supplementary material 1 (DOCX 5025 kb)
