# Supplementary material for: Impairments in contractility and cytoskeletal organisation cause nuclear defects in nemaline myopathy
Source: Acta Neuropathol. 2019 Jun 19;138(3):477–95. doi: 10.1007/s00401-019-02034-8 (PMC6689292; doi:10.1007/s00401-019-02034-8)
Supplement: Supplementary file 1 — Supplementary material 1 (DOCX 5025 kb) [file 401_2019_2034_MOESM1_ESM.docx]

**Supplementary figures**


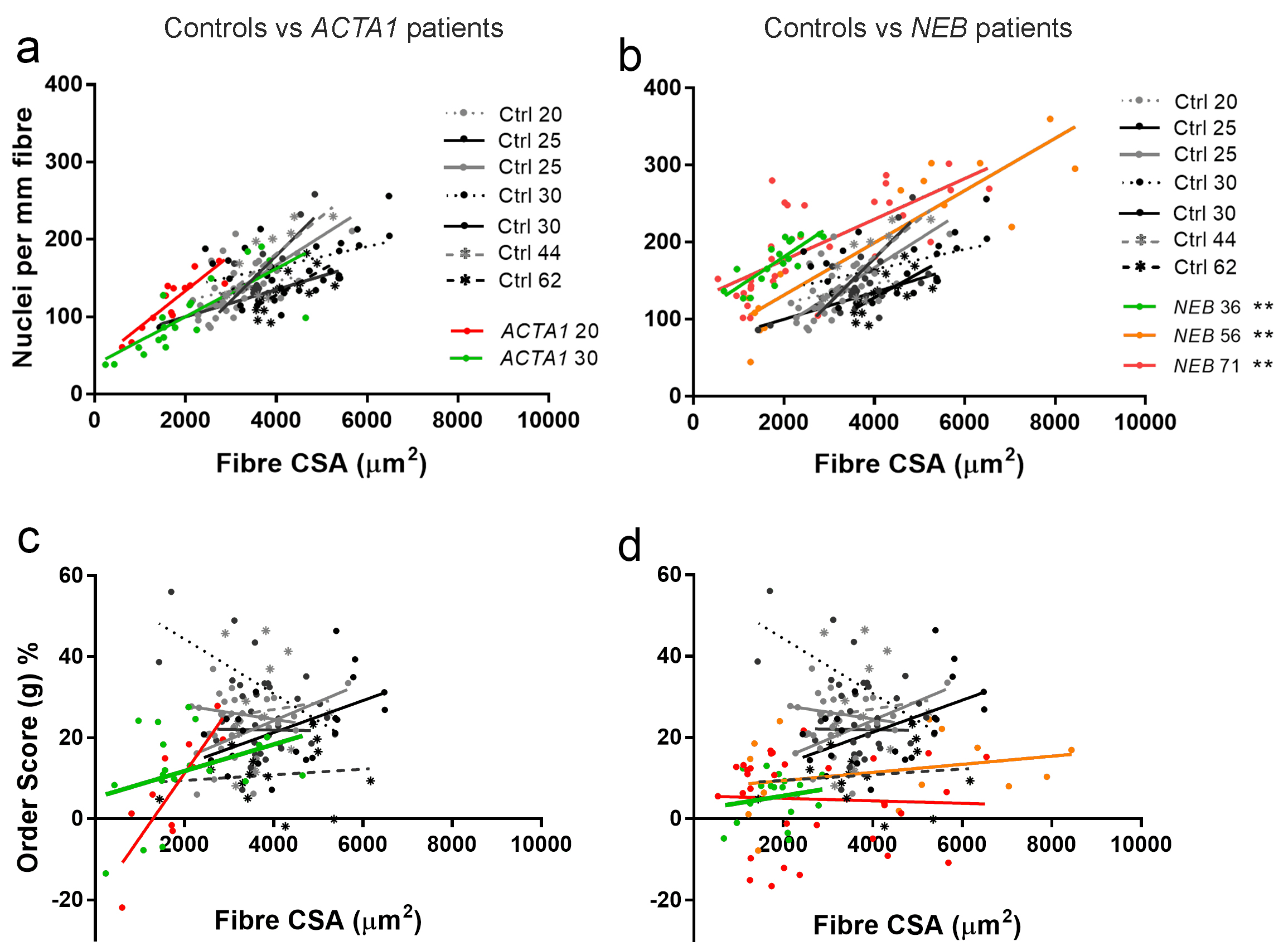


**Fig S1. Relationship between nuclear organisation and muscle fibre size (related to Fig 1).** Healthy control subjects and patients are denoted with their mutation and age. Individual data points represent an individual skeletal muscle fibre. **(a, b)** data is the same as that plotted in Fig 1e and f, but linear regression lines for controls are separated into each subject, rather than combined into a single line. Number of nuclei per mm of fibre in control subjects versus *ACTA1* patients **(a)** and control subjects versus *NEB* patients **(b)**. **(c, d)** Order score, an algorithm to assess the regularity of nuclear spacing; a lower score indicates more irregular spacing and more nuclear clustering. Order score for control subjects versus *ACTA1* patients **(c)**; and control subjects versus *NEB* patients **(d)**. For most controls and patients, either no correlation, or a weak positive correlation between fibre CSA and order score was observed. One patient (*ACTA1 20*) showed a positive correlation (R^2^ = 0.68), indicating that larger fibres tended to be more ordered than small, although one control (*Ctrl 30*) showed a negative correlation (R^2^ = 0.47), suggesting the reverse relationship.


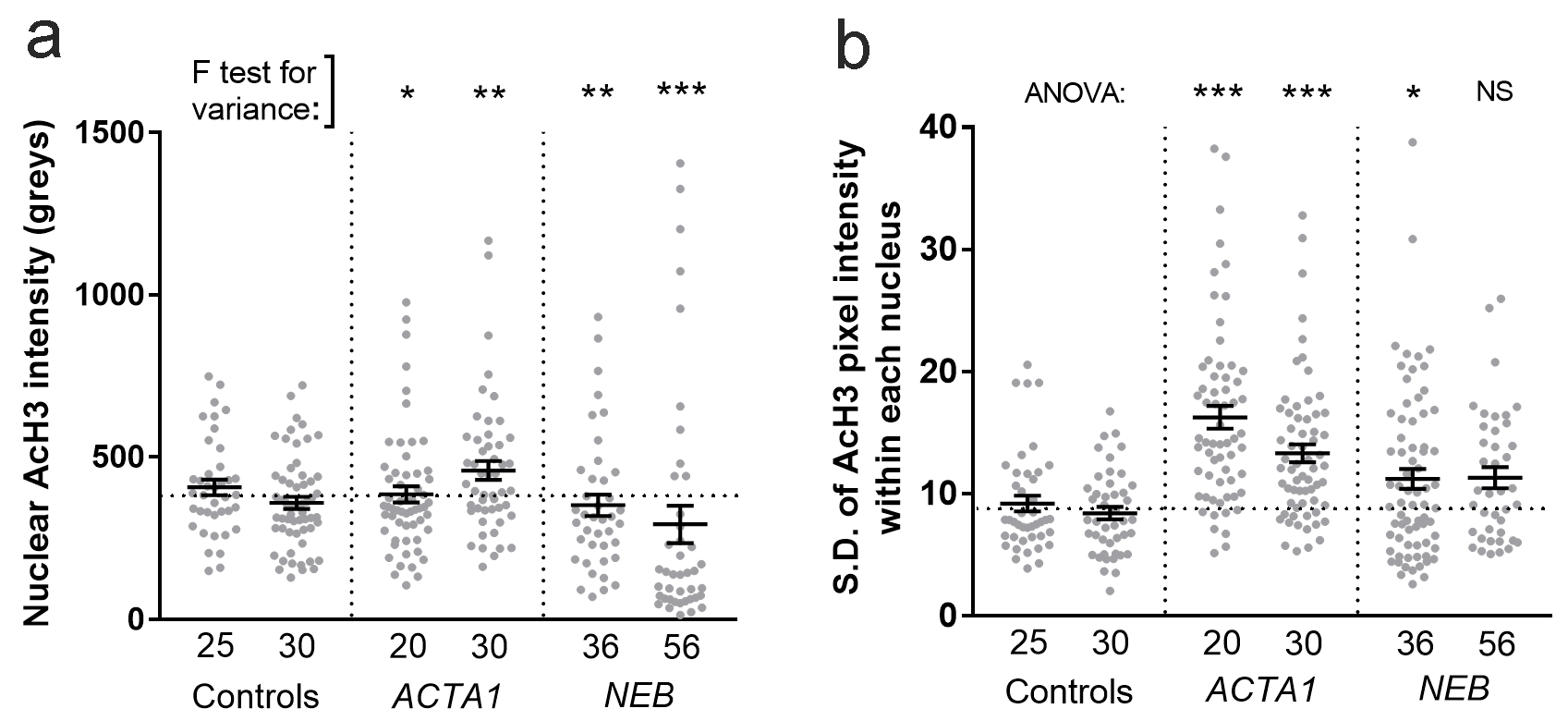


**Fig S2. Altered chromatin organisation in patients with nemaline myopathy (related to Fig 3i-k).** **(a)** Mean acetylhistone H3 pixel intensity per nucleus (one data point per nucleus measured); F test for variance indicates that the variation in staining intensity between nuclei is significantly greater in patients than controls. **(b)** Standard deviation of pixel intensity within each nucleus, as a measure of staining variability within the nucleus (one data point per nucleus measured); patients frequently have more variable staining within each nucleus, possibly indicating irregularly packed regions of chromatin. 50+ nuclei were observed per subject across ~9 fibres, mean +/- SEM. One-way ANOVA with Tukey post-correction and a random effect algorithm (to account for hypothetical inter-individual differences that might exist between controls) was used to compare each patient with controls. * (P<0.05), ** (P<0.01), *** (P<0.001).


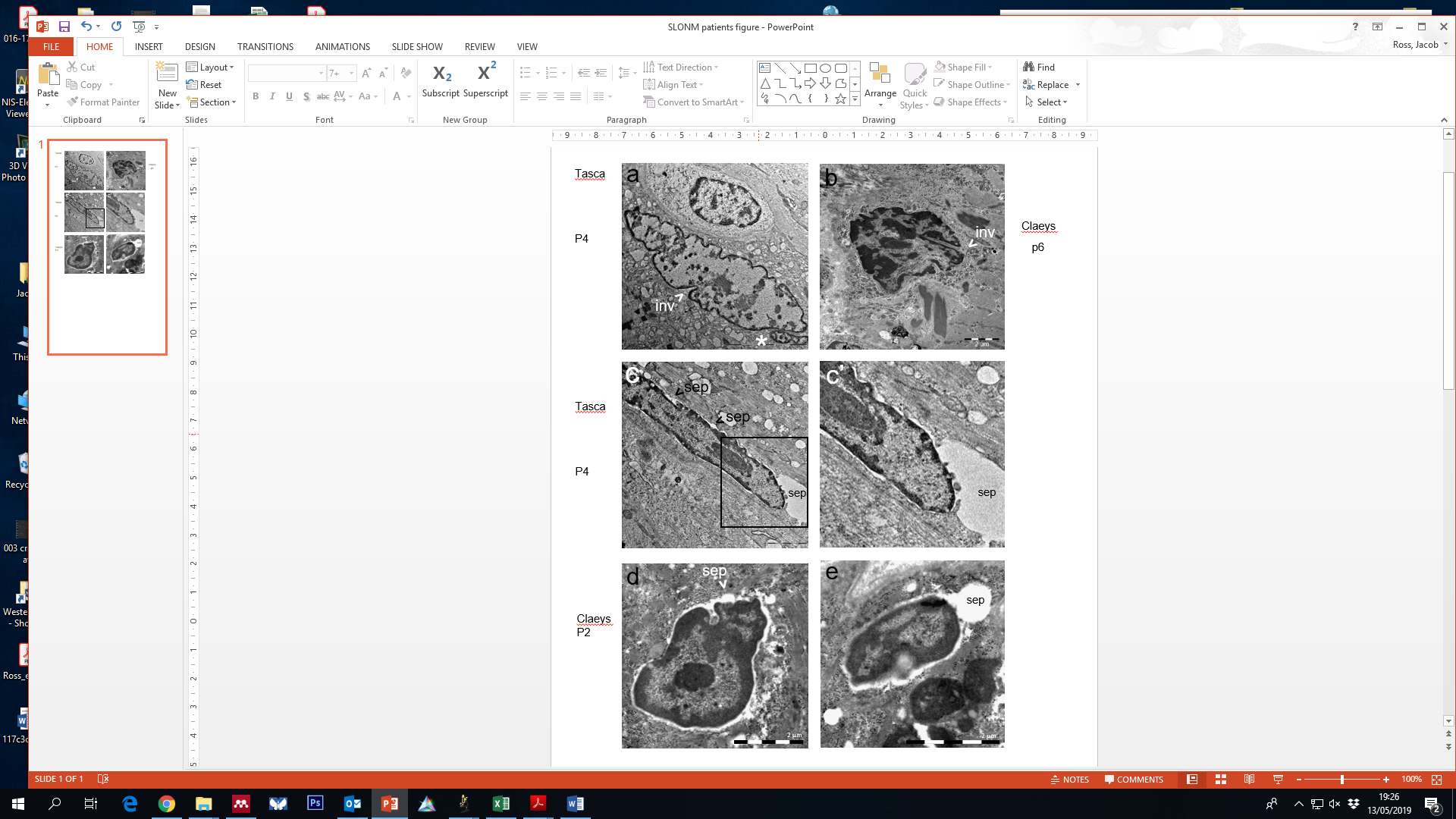


**Fig S3. Transmission electron microscopy of skeletal muscle biopsies of patients with sporadic late onset nemaline myopathy (SLONM).** See **Table 3** for patient details. **(a)** myonucleus from patient SLONM 1, showing invaginations (“inv”). In addition, a small fragment of nucleus is present in the bottom right corner (*), which may represent a separate nuclear entity or a lobulation of the same nucleus**. (b)** myonucleus from patient SLONM 5 with dense chromatin/high levels of heterochromatin, and a large invagination. **(c)** myonucleus from patient SLONM 1 with several regions of separation (“sep”) between inner and outer nuclear membranes, of varying width. **(c’)** is a magnification of the boxed region in **(c)**. **(d)** and **(e)** myonuclei from patient SLONM 3 showing variable separations between inner and outer nuclear membranes. In addition, chromatin is highly condensed in these nuclei. A semi-quantitative analysis of electron microscopy findings in SLONM patients are shown in **Table 5**.


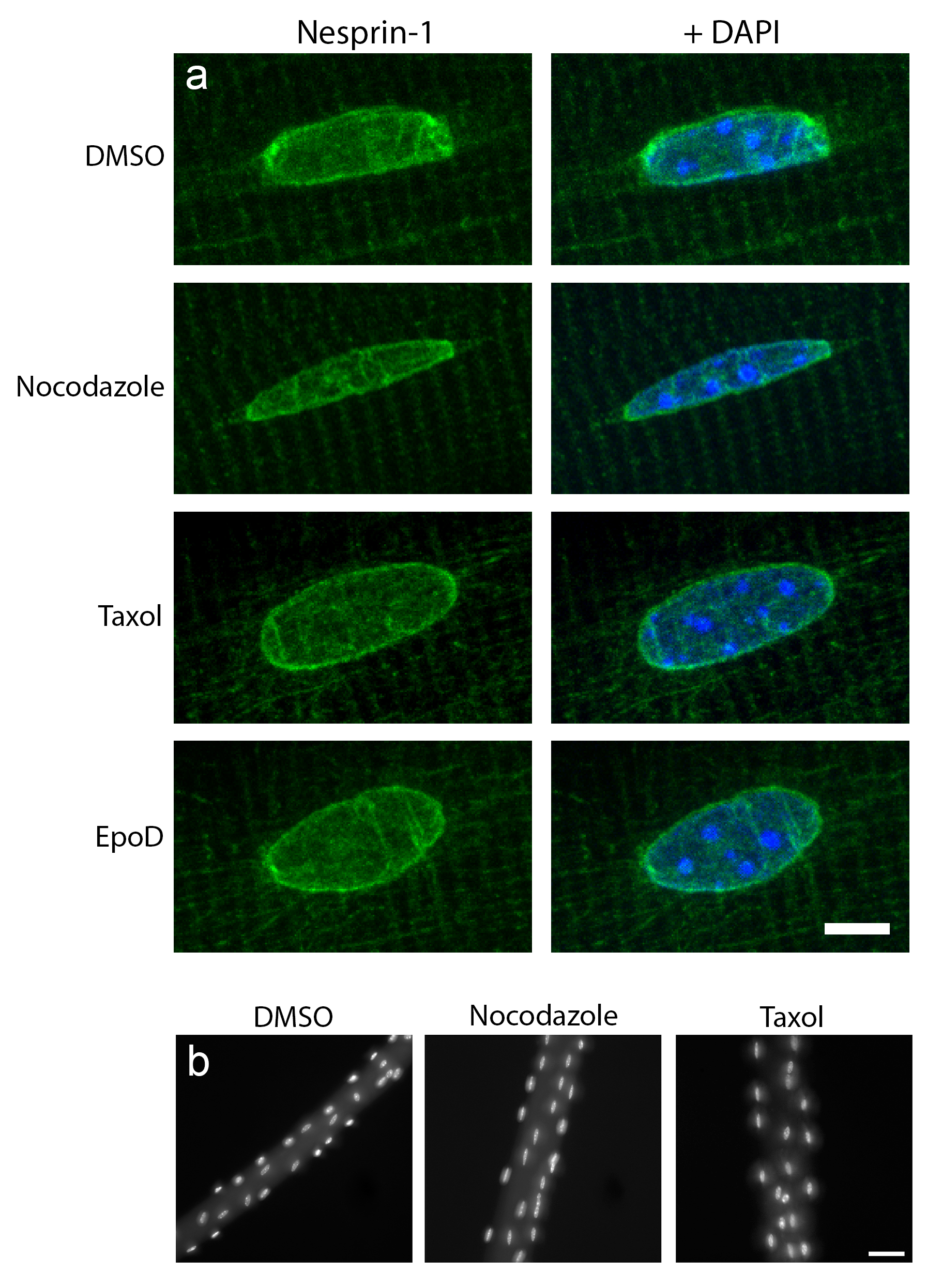


**Fig S4. Nesprin-1 localisation and myonuclear spacing is unaffected by microtubule perturbations (related to Fig 7).** **(a)** Typical myonuclei from mouse skeletal muscle fibres treated overnight with vehicle (DMSO), nocodazole, taxol or epothilone D. Nesprin-1 staining (green) and DAPI (blue). The localisation of nesprin-1 was not markedly affected by treatment with microtubule perturbing drugs. ~50 nuclei were observed per condition across 2-3 separate experiments. **(b)** Representative DAPI-stained images of muscle fibres treated with DMSO, nocodazole or taxol. No overt alterations to myonuclear spacing were observed in response to the drugs, even after 72 hours (~20 fibres observed across 2-3 experiments). Scale bars: 5μm (A); 50μm (B).
